# Trends in socioeconomic inequalities in anthropometric status in a population undergoing the nutritional transition: data from 1982, 1993 and 2004 pelotas birth cohort studies

**DOI:** 10.1186/1471-2458-12-511

**Published:** 2012-07-09

**Authors:** Alicia Matijasevich, Iná S Santos, Ana M B Menezes, Aluísio J D Barros, Denise P Gigante, Bernardo L Horta, Fernando C Barros, Cesar G Victora

**Affiliations:** 1 Postgraduate Programme in Epidemiology, Federal University of Pelotas, Pelotas, RS, Brazil; 2 Postgraduate Programme in Health and Behaviour, Catholic University of Pelotas, Pelotas, RS, Brazil

**Keywords:** Socioeconomic factors, Health status disparities, Cohort studies, Child nutrition, Overweight, Stunting

## Abstract

**Background:**

Socioeconomic inequalities in child nutrition may change rapidly over time, particularly in populations undergoing the nutrition transition. Yet, the few available studies are repeated cross-sectional surveys. By studying three prospective birth cohorts in the same city over a period of more than two decades, we describe secular trends in overweight and stunting at different ages, according to socioeconomic position.

**Methods:**

Population-based birth cohort studies were launched in the city of Pelotas (Brazil) in 1982, 1993 and 2004, with follow-up visits at twelve, 24 and 48 months. Children were weighed and measured at every visit. Z-scores of length/height-for-age and body mass index-for-age were calculated using the WHO Child Growth Standards. The slope and relative indices of inequality, based on family income quintiles, were estimated for each follow-up visit.

**Results:**

Between the 1982 and 2004 cohorts, stunting among four-year-olds declined (from 10.9% to 3.6%), while overweight increased (from 7.6% to 12.3%). In every visit, stunting prevalence was inversely related to income. Both absolute and relative inequalities declined over time; among four-year-olds stunting dropped from 26.0% in the 1982 cohort to 6.7% in the 2004 cohort in the poorest group, while in the richest group stunting prevalence dropped from 2.7% in 1982 to 1.1% in the 2004 cohort study. The secular trend towards increased overweight was evident for four-year-olds, in almost all socioeconomic groups, but not among one and two-year-olds. Among four-year old children, overweight prevalence increased in all income quintiles, by 130% in the middle-income group, 64% in the poorest and 41% in the richest group.

**Conclusions:**

The decline in stunting is remarkable, but the increase in overweight among four-year olds – particularly among the poorest and the middle-income groups– requires concerted efforts to prevent the long term consequences of child overweight.

## Background

The double burden of malnutrition in populations undergoing economic development includes both undernutrition and overweight [1]. Linear growth retardation or stunting is a measure of long-term undernutrition, which is generally caused by a combination of poor nutrition, infectious diseases and suboptimal child care [2,3]. Stunting in childhood has been shown to negatively affect human capital in the short and long-term, including lower intellectual performance, reduced work capacity and poor reproductive performance [4,5]. Suboptimal growth in early life has also been associated with chronic conditions, such as cardiovascular disease and type 2 diabetes [6]. Stunting affects approximately 195 million children under five years old in the developing world, with the highest prevalence found in Africa and Asia (40% and 36%, respectively) [7]. In the last decades stunting prevalence declined in most developing countries; trend analyses based on 80 countries showed that prevalence of stunting dropped from 40% in 1990 to 29% in 2008 in the developing world [7].

Conversely, a different picture emerges when it comes to overweight, a condition associated with excess nutrient and energy intake relative to expenditure. Overweight and obesity increase the risk of a number of chronic diseases, which can lead to reduced quality of life and premature death [8,9]. In addition, there is evidence of tracking of overweight, with several studies showing that overweight children tend to remain so during adolescence [10,11].

The association between socioeconomic position (SEP) and nutritional status is well established, but it can go in different directions according to the level of socioeconomic development of the population. It also varies by gender. McLaren [12] analyzed the socioeconomic patterning of weight across societies in various stages of socioeconomic development. In general, both men and women from the lowest SEP have a higher likelihood of obesity in developed countries while in low and middle-income countries higher SEP is frequently associated with excessive weight [12,13]. Similar associations have been reported for children [14]. In high-income populations, child obesity prevalence is directly related to poverty [15,16]. The association between SEP and overweight/obesity among children from low and middle-income countries is less well studied. Wang [17] showed that while in China high socioeconomic children were at high risk of obesity, in Russia, both low-income and high-income groups were at an increased risk of obesity compared to the medium-income group. However, recent findings from Hong Kongs population-representative “Children of 1997” birth cohort found no consistent association between family or neighborhood SEP and childhood adiposity [18].

Much of the existing literature on socioeconomic inequalities in childhood nutrition relies on cross-sectional data, and reports on relatively wide age ranges, usually children under five years. The present analyses are based on data from three population-based cohort studies from a middle-income country - the 1982, 1993 and 2004 Pelotas cohorts – with the main objectives of describing secular trends in overweight and stunting at the ages of one, two and four years, and assessing how the association between malnutrition and socioeconomic status is evolving over time.

## Methods

### Research setting and study design

Pelotas is located in Southern Brazil, with a population of about 340,000 inhabitants, 93% of them living in the urban area (2000 Brazilian Demographic Census, IBGE). More than 99% of all birth deliveries take place in hospitals. During the years of 1982, 1993 and 2004, birth cohort studies representing all births to mothers residing in the urban area of the city of Pelotas, in Southern Brazil, were carried out entailing primary data collection and using much of the same methodology (6011, 5304 and 4287 births in 1982, 1993 and 2004, respectively). Eligible subjects for the perinatal study included all livebirths, and stillbirths weighing at least 500 g. The non-response rate at recruitment in the three cohorts was below 1%. A detailed description of the methodology is given elsewhere [19-21]. Soon after delivery, mothers were interviewed using a pre-tested structured questionnaire and their newborns were examined by specially trained field workers under the supervision of a pediatrician (Questionnaires of the 1982, 1993 and 2004 are available at http://www.epidemio-ufpel.org.br/site/content/estudos/index.php).

Children whose mothers lived in the urban area of Pelotas were visited at home at different points in time. On each occasion, mothers were interviewed and their children were weighed and measured by trained field workers. In the 1982 cohort, all children who were born from January to April 1982 were sought at the age of one year (1457 infants, 79% follow-up rate) and the whole sample was sought at approximately two and four years of age (4934 and 4742 children with follow-up rates of 87% and 84%, respectively). In the 1993 cohort, the sample included all low birthweight children plus 20% of the remaining (1460 and 1450 children at one and four years old, with follow-up rates of 93% and 87%, respectively). In the 2004 cohort study, attempts were made to see all children when they were one, two and four years old (3907, 3869 and 3799 children, with follow-up rates of 94%, 94% and 92%, respectively). Stillbirths were not included in the analyses of nutritional indicators at birth.

### Anthropometric measurements

In the three cohorts, birthweight was measured by hospital staff with 10-g precision pediatric scales that were regularly calibrated by the research team. Supine length measurements were taken using AHRTAG infantometers (AHRTAG baby length measures, London) [22].

At each follow-up, anthropometric measurements were performed by trained interviewers with the children dressed in underwear and barefoot. When clothing was worn, these items were noted and had their weights subsequently deducted from the child’s measured weight. In the 1982 and 1993 cohort study, children were weighed using Salter CMS mechanical scales with 25 kg maximum and 100 g precision. Tanita electronic scales with 150 kg maximum and 100 g precision were used in the 2004 cohort study. In the three studies, scales were calibrated on a weekly basis using standard weights. Recumbent length (children ≤24 months of age), and standing height (48 months of age) were measured using AHRTAG portable infantometers with 1 mm precision, custom built for these studies.

### Variables

Based on the collected data, we calculated *z*-scores for length/height-for-age, weight-for-age, weight-for-length/height, and body mass index-for-age according to the growth curves published by the World Health Organization in 2006 [23]. Prevalence of stunting, underweight and wasting were defined as the percent of children with z-scores of length/height-for-age, weight-for-age and weight-for-length/height below −2, respectively. Overweight was defined as the percent of children with more than 2 z-scores of body mass index (BMI) for their age [23,24].

The WHO standard was chosen over the International Obesity Task force (IOTF) definition because the latter starts at the age of two years [25]. In addition, the IOTF could not be used for the two year follow-up of the 1982 and 2004 Pelotas cohorts because many children were below 24 months (mean age 19.4 months and 23.9 months in the 1982 and 2004 cohort study, respectively). A separately analysis using IOTF cut-offs for overweight and obesity for the four year follow-up of the three birth cohorts is provided as (Additional file 1: Table S1 and Additional file 2: Table S2).

To assess trends in socioeconomic inequality, family income during the month prior to the child’s birth was used as a socioeconomic position (SEP) indicator. All comparisons are based on quintiles of family income.

### Statistical analyses

We used the *χ*2 test to compare the prevalence of nutritional indicators (underweight, stunting, wasting and overweight) at each follow-up between the Pelotas cohort studies. When appropriate, we also performed tests for linear trends over the study period. Analyses of the 1993 cohort were weighted to correct for the over-sampling of low birthweight babies.

The slope index of inequality (SII) and relative index of inequality (RII) were estimated to measure inequalities in stunting and overweight across categories of family income in each cohort study [26,27].

The SII is derived via regression of mean health outcome within a particular social group on the mean relative rank of social groups. We kept our socioeconomic indicator constant in the three cohorts using quintiles of family income. By definition, each quintile included approximately 20% of the cohort, and midpoints of the quintile categories were calculated (approximately 0.1, 0.3, 0.5, 0.7 and 0.9 for the five quintiles). The SII was then obtained by regressing stunting and overweight prevalence on the midpoint score for each category. The slope of the regression line represents the absolute difference in the nutritional indicator between the highest (score of 1) and the lowest (score of 0) values of the socioeconomic indicator.

To estimate relative inequalities in stunting and overweight in the three birth cohort studies, logistic regression analysis was used to calculate relative indices of inequality (RII) [28]. As for the SII, the midpoints of the income quintiles constituted the independent variable in the analysis. The regression coefficient and its standard error were subsequently used to calculate the odds ratio with 95% confidence intervals. This odds ratio is known as the RII. The larger the RII the greater the degree of inequality across the socioeconomic hierarchy.

For each measure (SII or RII) and each cohort, the results were always for the comparison of the extremes – the highest compared with the lowest SEP position (that is, comparing the most with the least advantaged).

For each time assessment we calculated p-values from heterogeneity tests to assess whether the coefficients for the SII and RII were different across the three cohort studies. All analyses were performed with Stata software version 11.0 (StataCorp LP, College Station, Tex).

### Details of ethics approval

The study protocol of 1982, 1993 and 2004 Pelotas cohort studies was approved by the Medical Ethics Committee of the Federal University of Pelotas, affiliated with the Brazilian Federal Medical Council.

## Results

### Secular trends in under and over-nutrition prevalence

The prevalence of underweight, stunting, wasting and overweight, and the numbers of children with anthropometric data in each follow-up are provided in Table 1. Missing data for nutritional variables was below 5% in the three cohort studies. The frequency of nutritional deficits tended to decrease over time, with stunting showing the most marked declines (29%, 64% and 67% at one, two and four years, respectively), while the prevalence of overweight increased. The exceptions to this pattern included underweight and stunting at birth, both of which were highest in 2004; overweight at one and two years of age, which did not show significant increases over time; and wasting at two and four years, which remained stable at very low levels. Because wasting and underweight are relatively rare in this population, the following analyses will focus on stunting and overweight.

**Table 1:** Time trends in the prevalence of nutritional indicators in the Pelotas cohort studies

**Indicators**	**1982% (n)**	**1993% (n)**	**2004% (n)**	**p**^**a**^
***At birth***	5914	5249	4231	
Underweight	7.1 (418)	7.8 (405)	7.9 (332)	0.118
Stunting	(−)	9.3 (476)	12.4 (513)	<0.001^b^
Wasting	(−)	3.6 (179)	1.3 (50)	<0.001^b^
Overweight	(−)	2.2 (112)	2.5 (105)	0.261 ^b^
***1 year***	1457	1361	3907	
Underweight	4.0 (58)	2.6 (65)	2.2 (86)	0.001
Stunting	8.5 (123)	9.2 (182)	6.0 (232)	0.001
Wasting	2.1 (30)	0.7 (17)	0.6 (25)	<0.001
Overweight	6.9 (101)	10.8 (129)	8.9 (347)	0.107
***2 years***	4939		3869	
Underweight	3.1 (154)	(−)	2.0 (77)	0.001^b^
Stunting	13.9 (687)	(−)	5.0 (192)	<0.001^b^
Wasting	0.8 (37)	(−)	0.8 (29)	0.995^b^
Overweight	8.5 (418)	(−)	8.3 (320)	0.781^b^
***4 years***	4742	1243	3799	
Underweight	2.3(109)	2.4 (39)	1.7 (64)	0.051
Stunting	10.9 (518)	5.3 (92)	3.6 (138)	<0.001
Wasting	0.3 (16)	0.4 (10)	0.6 (21)	0.130
Overweight	7.6 (358)	10.7 (121)	12.3 (463)	<0.001

(−) Data not available.

^a ^When 3 data points were available a *x*^2 ^test for linear trend was used.

^b ^For 2 data point a *x*^2 ^test for heterogeneity was employed.

### Secular trends in the socioeconomic patterning of stunting and overweight

In every comparison in the three cohorts, stunting was more frequent among the poorest compared to the richest children (Table 2). Absolute socioeconomic inequalities measured by the slope index of inequality were negative (reflecting lower prevalence among the rich than among the poor), but became closer to the null value over time. Relative inequalities, assessed by the relative index of inequality, were lower than one, reflecting lower prevalence of stunting among the richest than among the poorest, and also seemed to decline over time (however, p-values for heterogeneity tests were non significant). When trends in each income group are analyzed, the only clear reduction in stunting for one-year-olds was among the poorest (from 19.8% in 1982 to 8.1% in 2004); but at two and four years of age, there were significant declines in all social groups.

**Table 2:** Stunting prevalence per cohort and family income quintile, at 1, 2 and 4 years

**Cohort study**	**Stunting prevalence,%, per quintile**					**SII (95% CI)**	**RII (95% CI)**
	**Poorest**	**2**^**nd**^	**3**^**rd**^	**4**^**th**^	**Richest**		
***1 year***							
1982	19.8	10.6	6.0	5.5	3.6	−16.80 (−21.80; -11.82)	0.10 (0.04; 0.21)
1993	16.8	11.5	6.9	5.4	4.8	−18.52 (−24.84; -12.19)	0.19 (0.11; 0.34)
2004	8.1	9.1	5.7	3.8	3.1	−7.60 (−10.23; -4.98)	0.25 (0.15; 0.40)
*x*^*2 *^*test for linear trend*	*p < 0.001*	*p = 0.350*	*p = 0.752*	*p = 0.178*	*p = 0.525*	p < 0.001*	p = 0.125*
***2 years***							
1982	28.9	21.8	10.1	7.1	3.6	−32.04 (−35.34; -28.74)	0.05 (0.04; 0.07)
2004	8.8	8.4	3.8	2.4	1.5	−10.34 (−12.74; -7.93)	0.09 (0.05; 0.17)
*x*^*2 *^*test for heterogeneity*	*p < 0.001*	*p < 0.001*	*p < 0.001*	*p < 0.001*	*p = 0.007*	p < 0.001*	p = 0.205*
***4 years***							
1982	26.0	16.0	6.3	5.3	2.7	−27.79 (−30.83; -24.75)	0.04 (0.03; 0.06)
1993	11.8	4.6	5.5	2.3	2.6	−14.24 (−19.34; -9.15)	0.11 (0.05; 0.25)
2004	6.7	5.3	3.1	2.0	1.1	−7.21 (−9.30; -5.11)	0.11 (0.06; 0.21)
*x*^*2 *^*test for linear trend*	*p < 0.001*	*p < 0.001*	*p = 0.002*	*p < 0.001*	*p = 0.028*	p < 0.001*	p = 0.085*

CI = confidence interval; SII = slope index of inequality; RII = relative index of inequality.

* *x*^2^ test for heterogeneity to assess whether the coefficients of SII and RII are different across the three cohort studies.

**Figure 1:**
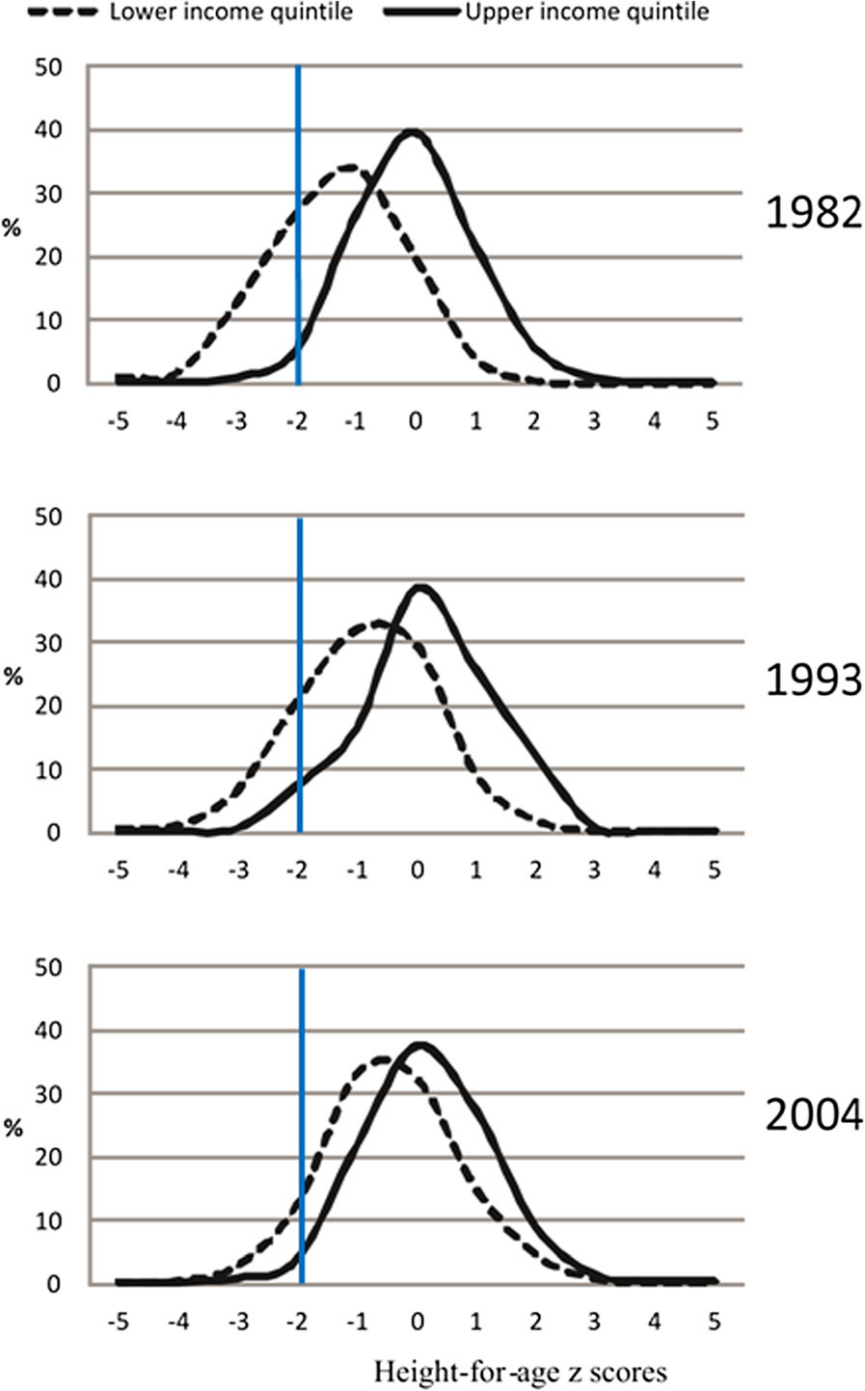
Height-for-age z score distribution for lower and upper income quintiles at 4 years of age.

Reductions in stunting were more marked between 1982 and 1993. Among four-year-olds, stunting among the poorest was reduced by nearly three quarters from 1982 to 2004. The exception to this pattern was the absence of a decline in relative inequalities between 1993 and 2004 at four years of age. Similar shifts can be observed in the height-for-age z-score (continuous variable) distribution over time. Height-for-age z score distribution for four-year-olds in the lower and upper income quintile are shown in Figure 1, demonstrating that the absolute gap between the poorest and richest children decreased over time. Similar patterns were observed for one- and two-year-olds (data not shown, available on request).

The secular trend towards increased overweight prevalence was evident for four-year-olds, in all socioeconomic groups (Table 3), but there were no clear trends for children aged one and two years. In the 1982 cohort, overweight was considerably more prevalent among the wealthy, in all follow-up visits and at all ages (almost 1.8 times, 1.5 and 2 times at one, two and four years, respectively). For one-year-olds, overweight nearly doubled over time among the poorest and among the middle-income group while remaining almost stable in the richest; as a result, the absolute gap decreased. Among two-year-olds the prevalence of overweight remained almost stable in all socioeconomic groups. Among four-year old children, overweight prevalence increased in all income quintiles, by 130% in the middle-income group, 64% in the poorest and 41% in the richest group. The most marked increase among the rich and among the middle-income group occurred between the 1982 and 1993 cohort (measured in 1986 and 1997, respectively), whereas for the poorest quintile the rapid increase took place between the 1993 and 2004 cohorts (measured in 1997 and 2008). Absolute differences increased between the 1982 and 1993 cohorts, and were reduced thereafter; similar patterns seemed to be observed for relative differences, however, confidence intervals overlapped.

**Table 3:** Overweight prevalence, per cohort and family income quintile, at 1, 2 and 4 years

**Cohort study**	**Overweight prevalence,%, per quintile**					**SII (95% CI)**	**RII (95% CI)**
	**Poorest**	**2**^**nd**^	**3**^**rd**^	**4**^**th**^	**Richest**		
***1 year***							
1982	5.0	7.9	4.4	7.8	9.2	3.93 (−0.69; 8.55)	1.85 (0.90; 3.80)
1993	12.9	9.9	8.0	14.4	9.1	−0.14 (−5.66; 5.38)	0.98 (0.52; 1.87)
2004	9.2	8.1	9.5	9.5	8.4	−0.12 (−3.29; 3.05)	0.99 (0.67; 1.46)
*x*^*2 *^*test for linear trend*	*p = 0.222*	*p = 0.862*	*p = 0.006*	*p = 0.763*	*p = 0.636*	p = 0.335*	p = 0.525*
***2 years***							
1982	7.2	6.2	8.5	9.9	10.5	5.05 (2.31; 7.80)	1.93 (1.35; 2.76)
2004	7.5	8.2	7.0	8.9	10.0	2.86 (−0.22; 5.94)	1.46 (0.97; 2.18)
*x*^*2 *^*test for heterogeneity*	*p = 0.800*	*p = 0.106*	*p = 0.224*	*p = 0.488*	*p = 0.767*	p = 0.298*	p = 0.321*
***4 years***							
1982	5.3	6.4	5.4	9.8	10.8	7.18 (4.52; 9.84)	2.84 (1.92; 4.20)
1993	6.0	10.1	8.7	13.5	16.0	12.69 (6.83; 18.54)	4.36 (2.18; 8.73)
2004	8.7	10.3	12.6	14.9	15.2	8.80 (5.09; 12.51)	2.27 (1.61; 3.22)
*x*^*2 *^*test for linear trend*	*p = 0.007*	*p = 0.003*	*p < 0.001*	*p = 0.001*	*p = 0.006*	p = 0.233*	p = 0.390*

CI = confidence interval; SII = slope index of inequality; RII = relative index of inequality.

* *x*^2^ test for heterogeneity to assess whether the coefficients of SII and RII are different across the three cohort studies.

**Figure 2:**
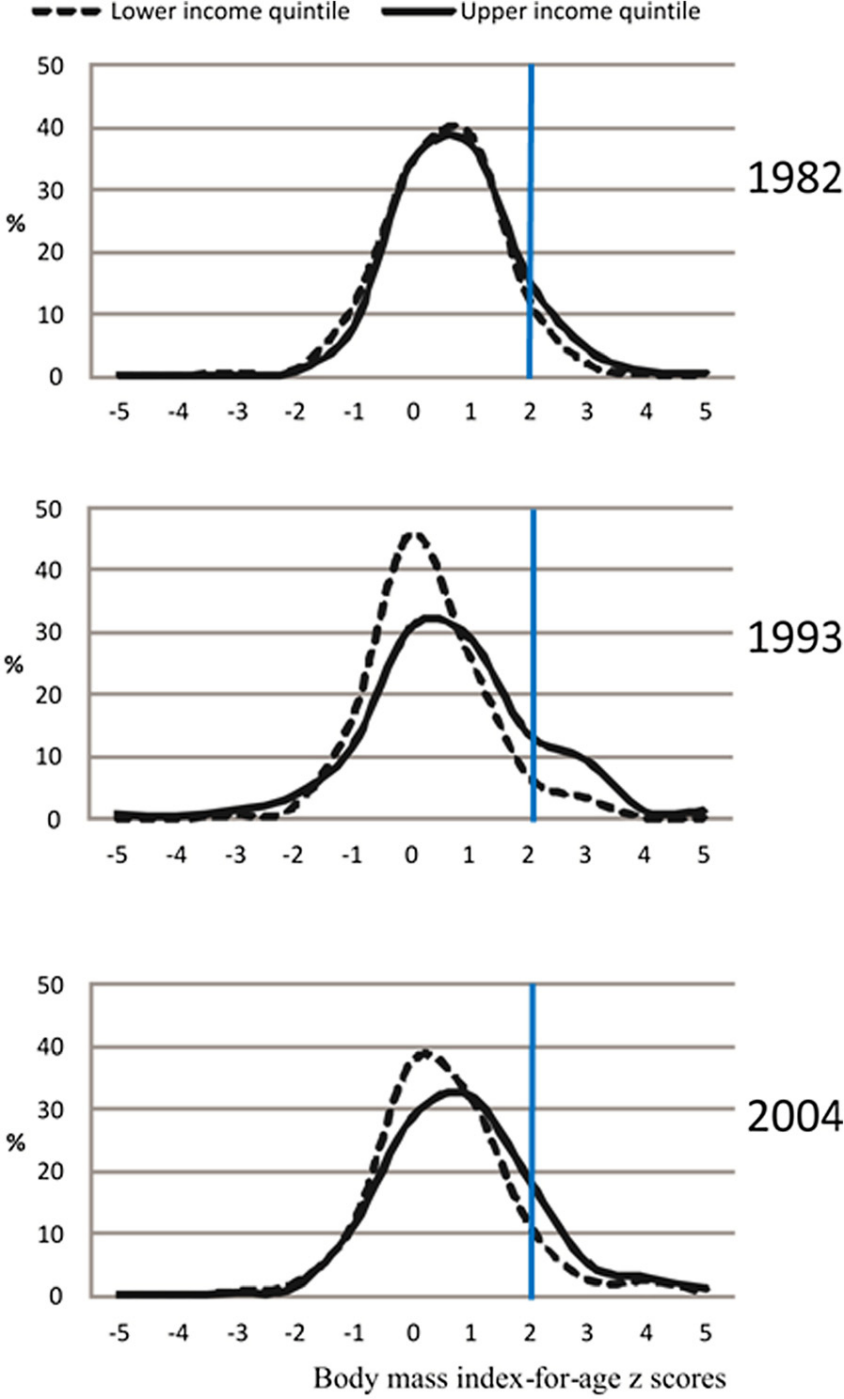
Body mass index-for-age z score distribution for lower and upper income quintiles among 4 year-olds.

The BMI-for-age distribution at four years of age (Figure 2) shows that in 1982 the curves were quite similar, with a small shift to the right in the rich, in spite of the poor being much shorter (Figure 1). Over time, the curves become more positively skewed, reflecting the increasing numbers of children with high BMIs (skewness of 0.376, 0.527 and 0.615 for four year old children from 1982, 1993 and 2004 cohort study, respectively). The 1993 curves appear to be bi-modal, and by 2004 one can note the presence of children with extreme obesity (BMI around 4 z-scores).

**Figure 3:**
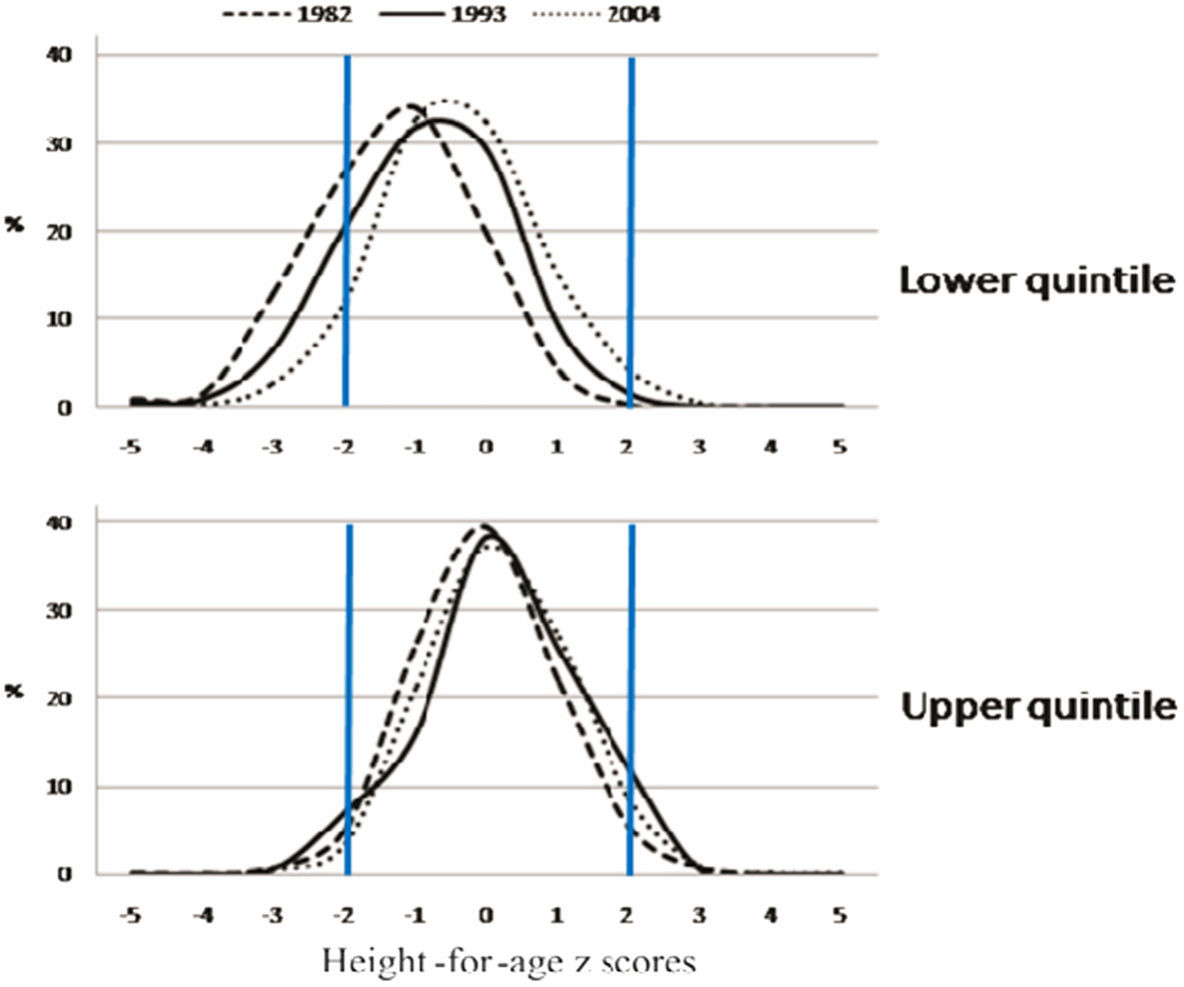
Trends in height-for-age z score for lower and upper income quintile among 4 year-olds.

The nutrition transition, reflected by the height-for-age z-score distribution in the extreme income categories among four year-olds is shown in Figure 3. The poorest children changed rapidly from 1982 to 1993, and less rapidly after that; mean (SD) height-for-age z-score values were −0.660 (1.112) in 1982, -0.330 (1.172) in 1993 and −0.148 (1.064) in 2004 cohort study. The richest children already had low prevalence of stunting in 1982, being close to the WHO growth standards. Patterns for the change in the whole BMI-for-age z-score distribution over time are not so clear (data not shown, available on request).

### Comparative analyses using IOTF cut-offs for overweight and obesity

Separate analyses were conducted, using IOTF cut-offs for overweight and obesity for the four year follow-up of the three birth cohort studies (Additional file 1: Table S1 and Additional file 2: Table S2). The results are similar to those obtained using the WHO reference. Between 1982 and 2004, overweight and obesity prevalence among four year old children increased 23% and 160% times, respectively (Additional file 1: Table S1). In the three cohorts, obesity was more frequent among the richest compared to the poorest children (Additional file 2: Table S2). Absolute socioeconomic inequalities were positive and relative indices were higher than one, reflecting higher prevalence of obesity among the richest than among the poorest. Absolute differences increased between the 1982 and 1993 cohorts, and were reduced thereafter; relative inequalities seemed to decline over time, however, confidence intervals overlapped. When trends in each income group were analyzed, although there were significant increases in obesity prevalence in all social groups, the highest increase was observed among the poorest children (almost five fold).

## Discussion

During the study period, the frequency of nutritional deficits was reduced, with stunting showing the most marked declines. In every comparison in the three cohorts, stunting was more frequent among the poorest compared to the richest. Both absolute and relative socioeconomic inequalities declined over time; among four-year-olds stunting was reduced by nearly three quarters in the poorest group from 1982 to 2004. On the other hand, the secular trend towards increased overweight was evident for four-year-olds in almost all socioeconomic groups, but there were no clear trends for children aged one or two years. Our results suggest that relative socioeconomic inequalities in overweight among four-year-olds appear to have been decreased mainly due to a proportionately larger increase of obesity among the poorest (64% increase among the poorest compared to 41% increase among the richest).

These three studies offer a unique opportunity for documenting the nutrition transition in a population from the same location and which belong to the same ethnic background. The comparisons rely on uniform modes of data collection (prospective information obtained among large population-based samples) combined with the use of similar questionnaires, standardised anthropometric measurements performed by highly trained fieldworkers, high follow-up rates and low frequencies of missing data (below 5%) for nutritional variables in the three birth cohort studies. Another advantage of our study over those using cross-sectional data is that it was possible to examine both cohort effects – i.e. changes in stunting and overweight over time comparing the same age group across cohorts, and age effects – i.e. comparing across ages within each cohort.

The one-year follow-up in the 1982 cohort, and the one and four-year follow-ups in 1993 cohort were based on subsamples rather than the whole cohorts. The 1993 subsamples were similar to the rest of the cohort in terms of socioeconomic position and maternal characteristics. Nevertheless, due to over-representation of low birthweight babies in the lower income groups we needed to weight all analyses of the 1993 cohort subsamples. In the 1982 cohort, children not included in the one-year subsample were born to poorer and less educated mothers than those followed up. It is unlikely that attrition or use of subsamples have introduced important biases in the present results.

There is debate about the accuracy of BMI as a measure for assessing adiposity in individual children [29]; however, it is widely accepted as a population estimate of obesity risk [29,30]. We opted to use BMI-for-age indicator to estimate child overweight in our population, following World Health Organizations (WHO) recommendations [23].

Levels and trends in stunting and overweight in Pelotas have to be placed in the international context. In South America, Guatemala, Peru, Honduras and Bolivia have the highest prevalence of stunting (54%, 30%, 29% and 22%, respectively). Brazils prevalence of stunting (7%) is almost the same as found in Argentina (8%), but higher than that of Chile (1%) [7]. In the 2004 cohort, stunting prevalence ranged from 4-6% depending on age. Most developing countries showed declines in the last decades [7]. In South America, overall stunting levels fell from 20.9% in 1990 to 13.1% in 2007 [31]. The decline in Brazil has been particularly rapid (from a prevalence of 37.1% in 1974–75 to 7.1% in 2006–7) [32], and our current findings are consistent with those of Brazil as whole, although the local prevalence remained below the national average.

Childhood overweight and obesity are assessed through different definitions, by different authors. However, studies are consistent in showing that their prevalence is increasing both in high-income [33] and low and middle-income countries [1,7]. Popkin *et al*[34]. compared trends in overweight prevalence (International Obesity Task Force, IOTF, criteria) among adults and children above five years of age, showing that in many countries - including the US, the UK, Australia, China and Brazil - child overweight is increasing at a faster rate than adult obesity. Wang & Lobstein [35] estimated that over 46% of school-age children will be overweight (IOTF criteria), and about one in seven children will be obese (IOTF criteria) in the Americas by 2010. In contrast, an analysis of trends in Brazil [32] did not find an increase in overweight among all under-five children over a 33-year period (proportions of under-five children whose weight-for-height was ≥2 z scores were 8.4% in 1989, 6.6% in 1996 and 7.3% in 2006–7). Because these national studies were cross-sectional and included children in broad age ranges (under three or under five years of age) they were unable to describe age patterns in the nutrition transition process. For example, we showed that overweight (percent of children with more than 2 z-scores of BMI-for-age) increased primarily among four year olds, but not among younger children. The national, cross-sectional analyses from Brazil may have missed this increase, because the increase in overweight is only happening for older under-fives, and these children only account for a small fraction of all under-fives.

Previous research in the social patterning of overweight in the 1982 and 1993 Pelotas cohort studies showed that among 11-year-old boys and girls there was a strong direct association between SEP and overweight. In the 1982 cohort, overweight at 18 years of age showed a positive association with SEP for males, and an inverse association among females [36]. This is in agreement with studies of Brazilian adults, where wealthy men tend to be fatter, and wealthy women thinner, than the rest of the population.

Few studies from low and middle-income countries have analyzed socioeconomic inequalities in childhood stunting or overweight status over time, and all of these have relied upon repeated cross-sectional designs. Analyses of national surveys in Mexico [37] and Brazil [32] showed a steady decrease in the rates of stunting for under-five children followed by a decrease in both absolute and relative socioeconomic inequalities. In India, even though in the 1992–2005 period there was a decrease in the rates of stunting among children less than three years of age, relative social disparities either widened or remained stable over time [38]. An analysis of 47 DHSs (Demographic and Health Surveys) that contained information on the nutritional status of children aged up to five years of four regions (sub-Saharan Africa, eastern Mediterranean, south and south-east Asia and Latin America and the Caribbean) showed that in almost all countries stunting disproportionately affected the poor, however, Latin America and the Caribbean region showed the largest socioeconomic inequalities [39]. Investigators identified three patterns of socioeconomic inequalities in stunting: “mass deprivation”, where stunting is highly prevalent within the majority of population while a small privileged class is much better off; “exclusion”, where the prevalence of stunting is relatively low in the majority of the population, but was much higher among the poor, and a third pattern, called “queuing” which shows an intermediate situation. Our results showed that socioeconomic inequalities in stunting in the Pelotas cohort studies have been showing the “exclusion” pattern since 1982. Hence programmes targeted at the poorest people are needed to continue reducing inequalities.

Less has been written about overweight/obesity trend inequalities among children from low and middle-income countries. A recent review showed that the prevalence of childhood overweight (percent of children with more than 2 z-scores of weight-for-height) tended to be higher among the rich than among the poor in Ghana, Sierra Leone, Tajikistan and the Dominican Republic [40]. Similar results were found among Mexican school-aged children [41]. In the same way, our results showed higher prevalence of overweight among the highest SEP group in almost every follow-up in the three cohort studies.

In the 22-year period between 1982 and 2004, there were major political and economic changes in Brazil. A remarkable change, in the field of politics, was the end of the military dictatorship in 1985, which was followed by periods of financial chaos until inflation was finally controlled in the early 1990s. The country experienced economic growth since 2000 and several programs targeting the poorest population groups were implemented. Inequalities in income distribution in the country persisted between 1982 and 1993, with the Gini income distribution index remaining at 0.60. However, a reduction to 0.57 was recorded in 2004, with a further drop to 0.55 by 2008 [42]. Brazil dropped from being the country with the highest income concentration in the world during the 1980s to a ranking of 14. All of these changes must have affected the health and nutrition situation of young children [43].

In spite of economic improvement in the country as a whole, the Pelotas region had slower growth than the rest of the country. In 1980, the per capita gross domestic product (GDP) of the city was equivalent to 81.4% of the average value for the state of Rio Grande do Sul, where it is located, and 93.6% of the average value for Brazil as a whole. In 2002, the per capita GDP of the city fell to 58% and 73.5% of the average value for the state of Rio Grande do Sul and Brazil, respectively [44].

Nevertheless, substantial improvements in maternal health and education - including decreased fertility, increased birth intervals, declining prevalence of smoking during pregnancy and higher levels of schooling – were observed in Pelotas during the course of the study period [45]. In contrast, there were negative changes in birth outcomes (the mean birthweight decreased by 37 g and preterm births increased, from 6.3% in 1982 to 14.7% in 2004) [46] which may explain the higher prevalence of stunting at birth in 2004 relative to 1993. Possible explanations for the negative trends in birth outcomes, such as inadequate quality of antenatal care and increased medicalisation of pregnancy and childbirth, including labour induction, caesarean sections and inaccurate ultrasound scans, are provided elsewhere [45].

The 1,000 day period between the onset of pregnancy and age two is a window of opportunity to fight against undernutrition [47]. This is the age range during which stunting tends to occur. Adequate nutrition, control of infectious diseases and appropriate child care are essential to promote optimal growth [48]. On the other hand, rapid weight gain in early life, particularly after the age of two years, is related to increased risk of some chronic diseases [5]. For preventing life-long obesity in children from middle-income populations such as Pelotas, adequate attention must be given to the period immediately following the first 1,000 days – mostly, to weight gain from two years onwards, which our data suggest to be the time when rapid gains are occurring in this population.

## Conclusions

In the present study, absolute and relative socioeconomic inequalities in stunting declined over time. Secular trends towards increase overweight were evident for four-year-olds in almost all socioeconomic groups – particularly among the poorest and the middle-income group -, but there were no clear trends for children aged one or two years.

Relative socioeconomic inequalities in overweight among four-year-olds appear to have been decreased mainly due to a proportionately larger increase of overweight among the poorest. Programmes targeted at these groups are needed to continue reducing socioeconomic inequalities in childhood nutrition.

## Competing interests

The authors declare to have no competing interests.

## Authors’ contributions

AM and CGV identified the research question, conducted the analyses and wrote the first draft of the article. ISS, AMM, DPG, BLH, AJDB and FCB contributed to the interpretation of the findings and the writing of the article. All authors read and approved the final manuscript.

## Pre-publication history

The pre-publication history for this paper can be accessed here:

http://www.biomedcentral.com/1471-2458/12/511/prepub

## Supplementary Material

Additional file 1 **Table S1. **Time trends in the prevalence of overweight and obesity among four year old children from the Pelotas cohort studies according to the International Obesity Task Force (IOTF) definition. Click here for file

Additional file 2 **Table S2. **Obesity prevalence according to the International Obesity Task Force (IOTF) definition, per cohort and family income quintile, at 4 years. Click here for file
