# Supplementary material for: Trends in socioeconomic inequalities in anthropometric status in a population undergoing the nutritional transition: data from 1982, 1993 and 2004 pelotas birth cohort studies
Source: BMC Public Health. 2012 Jul 9;12:511. doi: 10.1186/1471-2458-12-511 (PMC3490989; doi:10.1186/1471-2458-12-511)
Supplement: Additional file 1 — Table S1. Time trends in the prevalence of overweight and obesity among four year old children from the Pelotas cohort studies according to the International Obesity Task Force (IOTF) definition. [file 1471-2458-12-511-S1.docx]

**Web Table S1**. Time trends in the prevalence of overweight and obesity among four year old children from the Pelotas cohort studies according to the International Obesity Task Force (IOTF) definition

| Indicators | 1982  % (n) | 1993  % (n) | 2004  % (n) | p^a^ |
| --- | --- | --- | --- | --- |
|  | 4742 | 1243 | 3799 |  |
| Overweight | 13.1 (621) | 12.3 (155) | 16.1 (603) | <0.001 |
| Obesity | 3.2 (151) | 7.9 (100) | 8.3 (310) | <0.001 |

^a^  *x*^2^ test for linear trend
