# Supplementary material for: Trends in socioeconomic inequalities in anthropometric status in a population undergoing the nutritional transition: data from 1982, 1993 and 2004 pelotas birth cohort studies
Source: BMC Public Health. 2012 Jul 9;12:511. doi: 10.1186/1471-2458-12-511 (PMC3490989; doi:10.1186/1471-2458-12-511)
Supplement: Additional file 2 — Table S2. Obesity prevalence according to the International Obesity Task Force (IOTF) definition, per cohort and family income quintile, at 4 years. [file 1471-2458-12-511-S2.docx]

**Web Table S2**. Obesity prevalence according to the International Obesity Task Force (IOTF) definition, per cohort and family income quintile, at 4 years

| Cohort study | Obesity prevalence,%, per quintile | | | | | SII (95% CI) | RII (95% CI) |
| --- | --- | --- | --- | --- | --- | --- | --- |
|  | Poorest | 2^nd^ | 3^rd^ | 4^th^ | Richest |  |  |
| ***4 years*** |  |  |  |  |  |  |  |
| 1982 | 1.2 | 2.3 | 2.3 | 5.0 | 5.0 | 5.15 (3.39; 6.91) | 5.78 (3.12; 10.73) |
| 1993 | 3.5 | 5.1 | 4.8 | 11.8 | 12.5 | 9.89 (4.56; 15.22) | 3.98 (1.69; 9.38) |
| 2004 | 5.5 | 6.5 | 7.9 | 10.7 | 10.8 | 7.22 (4.11; 10.33) | 2.63 (1.73; 4.01) |
| *x^2^ test for linear trend* | *p<0.001* | *p<0.001* | *p<0.001* | *p=0.001* | *p=0.006* | *p=0.171** | *p=0.259** |

CI = confidence interval; SII = slope index of inequality; RII = relative index of inequality

* *x*^2^ test for heterogeneity to assess whether the coefficients of SII and RII are different across the three cohort studies
